# The relationship between deferred imitation, associative memory, and communication in 14-months-old children. Behavioral and electrophysiological indices

**DOI:** 10.3389/fpsyg.2015.00260

**Published:** 2015-03-16

**Authors:** Emelie Nordqvist, Mary Rudner, Mikael Johansson, Magnus Lindgren, Mikael Heimann

**Affiliations:** Department of Behavioral Sciences and Learning, Linköping UniversityLinköping, Sweden; The Swedish Institute for Disability Research, Linköping UniversityLinköping, Sweden; Linnaeus Centre HEAD, Linköping UniversityLinköping, Sweden; Department of Psychology, Lund UniversityLund, Sweden

**Keywords:** deferred imitation, infant, memory, event-related potentials, associative memory, communication

## Abstract

The present study combines behavioral observations of memory (deferred imitation, DI, after a brief delay of 30 min and after a long delay of 2–3 weeks) and electrophysiological (event-related potentials, ERPs) measures of associative memory, as well as parental reports of non-verbal and verbal communication in sixteen 14-months-old children. Results show that for DI, the children remembered the stimulus after the brief but not after the long delay. There was a clear electrophysiological response indicating associative memory. Furthermore, a correlation between DI and ERP suggests that both measures of memory (DI and associative memory) tap into similar mechanisms in 14-months-old children. There was also a statistically significant relation between parental report of receptive (verbal) language and the ERP, showing an association between receptive language skills and associative memory.

## Introduction

Studying early learning and memory development is challenging since young preverbal children cannot report what they have learnt or can remember. Thus, researchers have focused on developing behavioral and electrophysiological methods that can reliably measure memory and learning in infancy, and that are analogous to methods based on verbal report often used with older children or adults. Behaviorally, deferred imitation (DI) is considered a reliable method that taps into an early form of declarative memory (Meltzoff, 1995; Rovee-Collier et al., 2001; Jones and Herbert, 2006; Richmond and Nelson, 2007; Mullally and Maguire, 2014). Electrophysiological correlates of associative memory in response to presented stimuli can be studied without the need for overt response from the child (e.g., Torkildsen et al., 2008). In the present study, we combine these two methods along with parental report of non-verbal and verbal communication to investigate the relationship between early indices of declarative memory and associative memory in prelingual infants, as well as their association with communicative development.

Associative memory is a process by which a cognitive representation of the spatiotemporal relation between stimuli is formed (Newcombe et al., 2012) and is linked to the formation of declarative memories (Eichenbaum, 2004). DI relies on the ability to form representations of associations between actions and objects. Thus, the ability to form representations of associations is fundamental to memory. This forms the connection between the phenomena tapped by the electrophysiological and behavioral memory measures used in the present study. Associative memory in infants can be studied by repeatedly presenting two stimuli simultaneously, e.g., pictures (Heimann et al., 2013), or sound and picture (Torkildsen et al., 2008) and then measuring the response (e.g., the change in observed looking behavior or electrophysiological measures of brain activity) when one of the stimuli is changed (e.g., Reynolds and Guy, 2012). This study compares an electrophysiological measure of associative memory with behavioral measures of declarative memory reflected by DI with an observation-only design (Heimann and Meltzoff, 1996).

Deferred imitation is widely used in studies investigating memory development in infancy (e.g., Meltzoff, 1988a,b; Lukowski et al., 2005; Heimann et al., 2006; Jones and Herbert, 2006; Rovee-Collier and Cuevas, 2009). It is a relatively easily administered method that does not involve any verbal response as it only requires the child to imitate a modeled action-on-object after the action has been presented. Successful imitation, that entails memory of a novel act seen only once, is considered to tap into an early form of declarative memory (Rovee-Collier et al., 2001; Jones and Herbert, 2006; Rogers et al., 2008). DI is contingent on the child forming a representation of the action in relation to the object and retrieving it at a later time. Studies using DI of actions-on-objects have shown that the method is well-suited for measuring memory in pre-lingual children (e.g., Meltzoff, 1988a,b; Heimann and Meltzoff, 1996; Jones and Herbert, 2006), even in infants as young as 6 months of age, demonstrating that an early form of declarative memory is in place (Barr et al., 1996; Collie and Hayne, 1999; Heimann and Nilheim, 2004; Bauer et al., 2006).

Deferred imitation has been shown to reflect individual differences in memory development (for a summary see for example Jones and Herbert, 2006), probably reflecting variations in early age-related postnatal brain maturation (e.g., Heimann and Meltzoff, 1996). Findings to date indicate that memory encoding is faster for older infants than for younger infants, possibly reflecting increasing myelination during the first year of life (e.g., Howe, 2011). Older infants have also been found to have more representational flexibility than younger infants (e.g., Hayne et al., 2000; Jones and Herbert, 2006) and older infants retain information longer than younger infants; 6 months-old infants can recall events after a 24 h delay and 14 months-old infants have been shown to remember events after delays of up to several months (e.g., Meltzoff, 1995; Jones and Herbert, 2006). In addition, there are indications that, by the age of 12 months, infants can form representations of events that persist even longer. For example, results from an eye-tracking study by Kingo et al. (2014) suggested that 3-years-old children recalled a brief, unique event that took place when they were 12 months-old.

Early memory development is associated with communicative development and general cognitive abilities (Strid et al., 2006). For example, Heimann et al. (2006) showed that DI ability at 9 months predicted non-verbal communication at 14 months. Gathercole et al. (1999) showed that children with a larger vocabulary had better phonological memory. This pattern was stable from 4 years through adolescence. In addition, it has been suggested by Ullman (2004) that vocabulary depends partly on declarative memory. According to this view, declarative memory is assumed to subserve language; knowledge about words is suggested to be dependent on the same structures as knowledge about facts and events, i.e., declarative memory (Ullman, 2004). Some basic features of the DI procedure may thus be suggested to be present in learning to communicate with words or gestures; the child needs to be able to learn the word or gesture from someone else, associate it with meaning, and store it as a mnemonic representation for use at another time (Heimann et al., 2006).

The mechanisms or processes behind developmental change in memory early in life are yet to be completely described, along with the relation between memory development and other developing cognitive processes such as language. One way to investigate these mechanisms (Carver et al., 2000; Bauer et al., 2006; Norwood et al., 2014) is to combine behavioral observation methods (e.g., DI) with measures of brain activity [e.g., electroencephalography (EEG) or event-related potentials (ERPs)]. This integration of methods entails challenges but could also provide a more fine-grained study and description of early memory development (e.g., Bauer, 2006; Riggins et al., 2013; Norwood et al., 2014).

Electroencephalography/event-related potentials provides a non-invasive method of measuring brain activity during cognitive processing (for example memory encoding) and is thus of special value for studying preverbal infants and special populations since the method does not require any verbal or motor responses (de Haan, 2007). Work to date typically describes certain ERP components that are associated with early memory; including the negative central (Nc, see de Haan, 2007 for an overview). Assessments of the strength (amplitude) and timing (latency) of the components typically guide the interpretation of these responses (e.g., Snyder et al., 2002). The Nc is thought to reflect attention to/detection of a stimulus that is novel or salient to the child, and is often reported to decrease over stimulus repetitions. Thus, it can be used as a neural index of learning (e.g., Reynolds and Richards, 2005; de Haan, 2007). Here, an increase in amplitude of the Nc is assumed to reflect allocation of attention to novelty, while a decrease indicates familiarity as the stimuli are repeated and learned (e.g., de Haan, 2007).

Only a few studies have explicitly studied DI in relation to ERP measures in infants. Carver et al. (2000) showed that in 9-months-old infants, successful DI was associated with a stronger Nc response to novel than to familiar stimuli, reflecting novelty detection. These results have been confirmed by other studies using a similar approach (Bauer et al., 2003, 2006). In a previous study we found that associative memory as measured by the difference in Nc between the learning and test phases of the task correlated significantly with declarative memory measured with DI in 14-months-old children (Heimann et al., 2013). Although similar findings have been reported previously by both Carver et al. (2000) and Bauer et al. (2003) our study was unique in several ways: it used a much briefer behavioral learning phase (LP) during DI and allowed for no physical exploration of the objects used before recall. Thus, no motor (procedural) memory could have been formed. In addition, no verbal cue was provided upon demonstration or recall. Therefore, we were able to draw the conclusion that in our study, successful DI was contingent on the establishment of cognitive representations of associations between actions and objects and could not have been supported either by internal motor representations or external linguistic cues.

Hence, the present study builds upon our previous findings. Since DI partly depends on the ability to learn and remember associations (here between an action and an object) the main goal is to further investigate the relation between associative memory as reflected by ERP and declarative memory as reflected by the behavioral response of DI. The ERP paradigm is inspired by Torkildsen et al. (2009), where learning of associations between words and their referents in 20 months-old children were studied. In the present study, stimulus pairs (pictures of namable agents and objects) are presented repeatedly during the LP. In the test phase, expectations built up during the LP are violated when familiar agents are presented with objects that are either entirely unfamiliar or occurred previously combined with other agents. In the first instance the measured response may simply reflect a reaction to the novelty of the stimulus while in the second case it is more likely to truly reflect associative memory (e.g., Aggleton et al., 2012). Our measure of associative memory, as in our previous study, is the change in Nc between the learning and test phases.

The main aim of the present study was to investigate the relationship between behavioral measures of DI, electrophysiological measures of associative memory, and early communicative ability. Based on the results of our previous study, we predicted that there would be an increase in mean amplitude of the Nc between the LP and both test phase variations, indicating learning of paired associations. In addition, we predicted a decrease in Nc mean amplitude between the beginning and end of the LP, indicating learning of the picture-pairs during the LP. Further, we predicted that the change of Nc mean amplitude between the LP and both test phases, reflecting the ability to form associative memories, would correlate with DI after a brief delay (30 min) as in our previous study. We also wanted to investigate if the same change in Nc also predicted memory after a long delay of several weeks, as indicated by for example Carver et al. (2000). Further, since previous studies have reported a relationship between early communicative development and memory we also investigated to what extent a measure of early language co-varied with DI and associative memory reflected by ERP.

## Materials and Methods

### Participants

Sixteen children were included in the experimental group of the present study (5 boys and 11 girls). Their mean age was 14.07 months (SD = 0.90, range: 12.23–15.49) and mean birth weight was 3473.44 g (SD = 426). The participants were recruited via well-baby clinics, open day-care units for parents, and their children, and word of mouth. All children were born full-term (mean gestational age 39.81 weeks, range 38–42) and had no known medical or developmental problems. An additional 28 children were recruited to the study but were excluded from analysis because ERP data were of inadequate quantity or quality. This was due to illness (*n* = 1), unwillingness to wear the ERP cap (*n* = 9), too few segments of adequate quality to be included in the analysis due to, e.g., too many artifacts in recording or too many segments during which the child did not attend to the stimulus (*n* = 16), or ERP measures that on several variables exceeded 3 SD from the mean (*n* = 2).

Of the 16 children, eight were also included in our previous study (Heimann et al., 2013). Thus, the DI data from these participants have been used also for this study, however, the ERP data have been re-analyzed and are not identical to those reported in our previous results.

In addition to the experimental group, a comparison group of eight children (five boys) was used for measuring baseline performance on DI, mean age = 15.43 months, SD = 0.62; range: 14.59–16.14.

This study was approved by the Regional Ethical Review Board, Linköping, Sweden. All caregivers gave their informed written consent before the testing began.

### General Procedure

**FIGURE 1:**
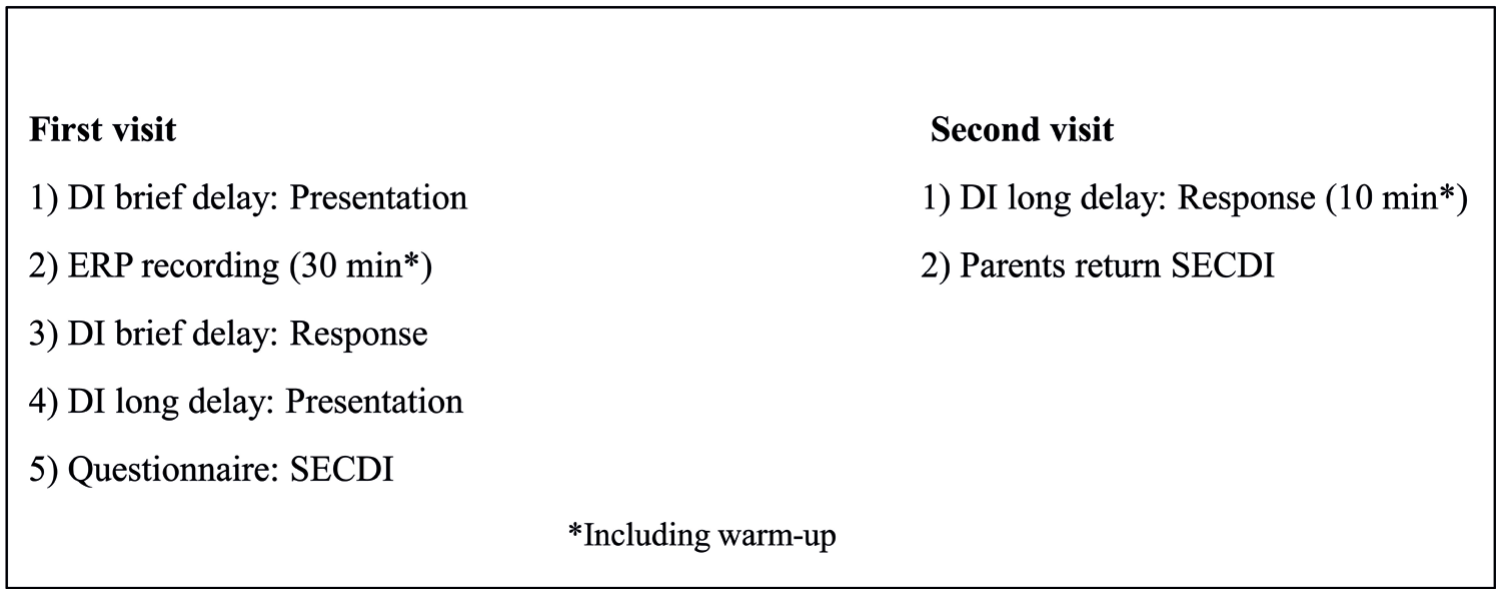
**Overview over general procedure**.

Eight of the participants in the experimental group made two visits to the laboratory with an interval of 2–3 weeks (*M* = 2.71 weeks, SD = 0.74), while the other eight (Heimann et al., 2013) visited the laboratory only once, see **Figure 1** for an overview. During the first visit, DI [presentation and response for the brief delay, (= 30 min between presentation and response) and presentation of DI with a long delay (2.71 weeks between presentation and response)] and ERPs were recorded. First, there was a warm-up session to familiarize the child with the lab and the experimenter. During this session, the experimenter also asked the parent about the child and his/her family, life, and development. Following this, the experimenter presented the actions-on-objects for DI with a brief delay. After presentation of the actions on objects for DI after the brief delay, the child and parent were escorted to another lab for the ERP recording. The ERP session took about 30 min, and then the participants were escorted back to the first lab for the rest of the DI procedure, including allowing the child to handle the objects and imitate the actions presented 30 min previously. Last, during the first visit, the actions on objects for DI after a long delay were also presented. The whole session was videotaped for later coding. At the second visit, the child was allowed to handle the objects presented at the first visit and imitate the corresponding actions. Other observations, not included in the present report, were also made at the second visit. The entire procedure was videotaped for later coding. Parents were instructed not to interact with the child during testing or to make comments on anything the child or experimenter did or said, even though the child sat in the parent’s lap during the testing. The comparison group visited the laboratory once and were tested for their spontaneous use of the objects at hand.

### Materials and Procedures

#### Behavioral Measures

##### Deferred imitation

For DI an observation-only procedure was used, where children were not allowed to handle the objects during or after the presentation, until it was time for DI, according to the procedure described by Meltzoff (1988a). One action-on-object at a time was presented three times during a period of about 20 s, and the order was counter-balanced over participants. Sets of three objects were used for each DI procedure. Objects were different for the two procedures:

###### Deferred imitation with a brief delay

The first object was a telescope-shaped cup that could be collapsed flat when pressed downward. The extended cup was presented and the target action was to press down on top of it with an open palm until it collapsed. The second object consisted of two 7.5 cm plastic tubes with plastic cylinders on one end of each tube. One of the tubes was slightly narrower than the other so that they could fit inside each other. The target action was to hold the cylinders at the end of each tube while they were joined together, and then to pull them apart with a definite motion. The third object consisted of a plastic cup and a short string of beads placed next to each other. The target action was to take the string of beads and place it in the cup.

###### Deferred imitation with a long delay

The first object was a rectangular black box with a button on the front. The target action was to take a pen from beside the box and press the button with the pen to make a ringing sound. The second object was a lamp that lit if pressed. The target action here was to light the lamp by pressing it with the forehead. The third item was an L-shaped wooden object consisting of two wooden blocks that were held together at a 90^∘^ angle by a hinge and the target action was to fold them together with the elbow.

After the delay, the child was allowed to handle the objects one at a time in the same order as they were originally presented. No verbal instructions were given by the experimenter during presentation or response sessions. The experimenter spoke only to get the participant’s attention by saying “look here” or the like.

##### The Swedish Early Communicative Development Inventories (SECDI)

Swedish Early Communicative Development Inventories (SECDI) is a Swedish version of the MacArthur Communicative Development Inventories (CDIs) and consists of two age-dependent versions (Fenson et al., 1994; Eriksson and Berglund, 1999). The CDI is a well-established questionnaire aimed at parents of young children who are asked to rate their children’s communicative skills. For the present study, the Swedish standardization is used. The age-dependent version used in the current study is designed to evaluate communicative skills in children between 8 and 16 months of age; “Words and gestures”. The inventory documents children’s understanding and production of words and sentences (maximum 385 words) along with production of communicative and symbolic gestures (maximum = 62). SECDI was given to the parents at the first session to fill in at home and the parents were asked to fill in the questionnaire as soon as possible after the session, and also to state the date on which the questionnaire was filled in. The rating scales are the frequency counts of each of the sections described above.

#### Electrophysiological Measures

##### Associative memory

A 30 cm × 40 cm computer screen displayed the stimuli for the ERP procedure. The participant sat in the parent’s lap about 1 m from the screen. The complete ERP session, including introduction to the lab environment, capping, impedance control, and lasted for about 30 min.

Sitting behind a partition so that distraction of the participant was minimized, the experimenter could manually control the stimulus presentation so that the pictures would only be shown when the child was alert and watching the screen. This was achieved by using a video camera to observe the participants during the ERP session. The videotapes were also later used for excluding segments during which the child was not attending to the screen.

Stimuli consisted of pairs of color clip art pictures that included one picture of an animal (e.g., a dog) and one of an inanimate object (e.g., ball). In the presentation of the picture-pairs, each animal was facing the inanimate object as if presenting it. During the LP, two animal-object pairs (e.g., dog-ball and deer-car) were presented alternately five times each (Presentations 1–5). During the subsequent test phase, two new pairs were presented twice each. These pairs always included each of the animals that had been presented during the LP but either paired with an object associated with a different animal during a LP (i.e., a recombination; Recomb) or with an object presented at no other time during the experiment (Novel). During the test phase, where the picture-pairs were presented pseudo-randomly, Recomb was always presented first, followed by Novel. This was followed by the LP pairs being presented once more as reminders. The test phase always ended with Novel followed by Recomb. The rationale behind the design of the learning and test phases was that new associations would be formed during the LP and violated during the test phase. For example, if dog-ball and deer-car were presented during the LP, dog-car (Recomb), and deer-tomato (Novel) might be presented during the test phase. While the electrophysiological response to Novel may simply reflect novelty detection, the response to Recomb is more likely to reflect violation of expectations generated by associative learning. The inter-stimulus interval was set to a minimum of 1000 ms and the duration of presentation of each picture pair was 1500 ms. The experimenter could pause the presentation of pictures if the child was not attending to the screen.

**FIGURE 2:**
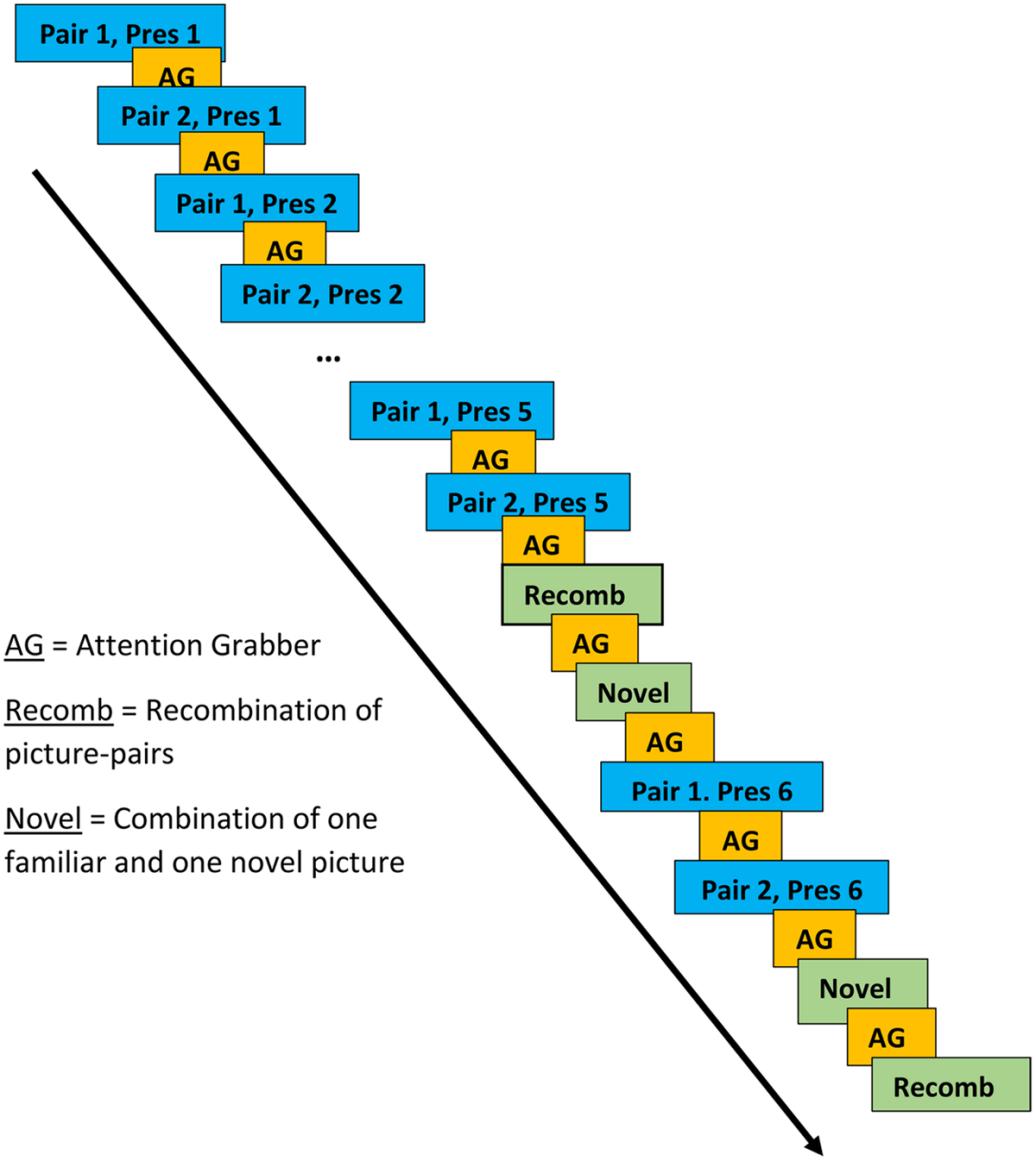
**Presentation of picture-pairs in learning and test phases.** Blue boxes represent the learning phase (LP), the yellow the attention grabber and the green boxes represent the test phase.

Presentations of the two picture pairs in the learning and test phases were separated by at least 3500 ms and presentation of the other picture pair (see **Figure 2** for a schematic representation of the various phases). An attention grabber in the form of a clown accompanied by a voice from the speakers saying “Look now” (Swedish: “Titta nu”) was presented in between each picture pair. During the test phase, each of the picture pairs from the LP was presented between the first and second presentations of the two new pairs. There was no indication to the participant of where each phase began or ended.

##### Recording of ERP

Continuous EEG was recorded by an Electrical Geodesics High Density sensor net containing 128 electrodes. EEG data were referenced to the vertex during recording and then re-referenced oﬄine to an average reference. The data was digitized at a sampling rate of 250 Hz and a 0.5–30 Hz bandpass filter was used. Impedances were kept below 50 kΩ. Segments of 1500 ms were extracted from the continuous EEG, including a 100 ms baseline. The data was baseline corrected and artifact detection was set to accept a max–min amplitude difference of 150 μV. Artifacts caused by eye blinks were corrected for using the algorithm adopted by Gratton et al. (1983). There was also a visual inspection for artifacts and drifts and these were subsequently corrected for using the interpolation method specified in the Net Station manual (Electrical Geodesics Inc, 2006). If more than 20% of leads in a trial were marked bad through artifact detection, that trial was rejected from further analysis unless some of the marked leads were in the neck area or some other place distant to the areas of interest.

In order for any particular participant to be included in further analysis, data for that participant had to contain a minimum of eight trials per condition (single conditions could contain seven but the majority of the conditions had to have at least eight valid trials), LP: *M* = 9.8 trials, range = 7–15; Recomb: *M* = 8.8 trials, range = 7–13; Novel: *M* = 9.8 trials, range = 7–14; Reminder: *M* = 9.8, range = 8–14. ‘Condition’ is referred to each of the presentations; Pres 1, 2, 3, 4, 5, 6, Recomb, Novel. All trials are averaged for each condition. There were 24 LP–test phase procedures and the mean number completed was 2.5.

### Data Analysis

#### Behavioral Measures

##### Deferred imitation with a brief delay

The scoring procedures and operational definitions were identical to the ones used in our previous study (Heimann et al., 2013); A yes/no code was used for registering whether the child performed the target action or not. A score of one was given for ‘yes’ and of zero for ‘no,’ yielding a range of scores of zero to three for the three actions on objects. A ‘yes’ was coded for the telescope-shaped cup if it was completely collapsed, for the plastic tubes if the two parts were successfully pulled apart, and for the plastic cup and short string of beads if the beads were lowered into the cup with no more than a third hanging over the edge of the plastic cup. A score was only given if the target action was performed within 30 s of the child first touching the object.

##### Deferred imitation with a long delay

The scoring procedure was the same as for DI with a brief delay. ‘Yes’ was coded in each case if the child managed to make the ringing sound by pressing the button on the black box with the pen, light the lamp with his/her forehead (or making an effort to do so with a definite motion leaning forward so that the distance between the lamp and the head is no more than 10 cm), or fold together the L-shaped wooden object with his or her elbow.

The first author and a research assistant who was blind to the hypotheses independently scored all of the observations for DI with a brief delay and with a long delay. The scoring made by the research assistant was made from film clips edited to only include the response periods, so that there was no clue of whether the actual clip was from the experimental group or the comparison group. The obtained agreement according to Cohen’s kappa was κ = 0.88.

#### Electrophysiological Measures

##### Associative memory

**FIGURE 3:**
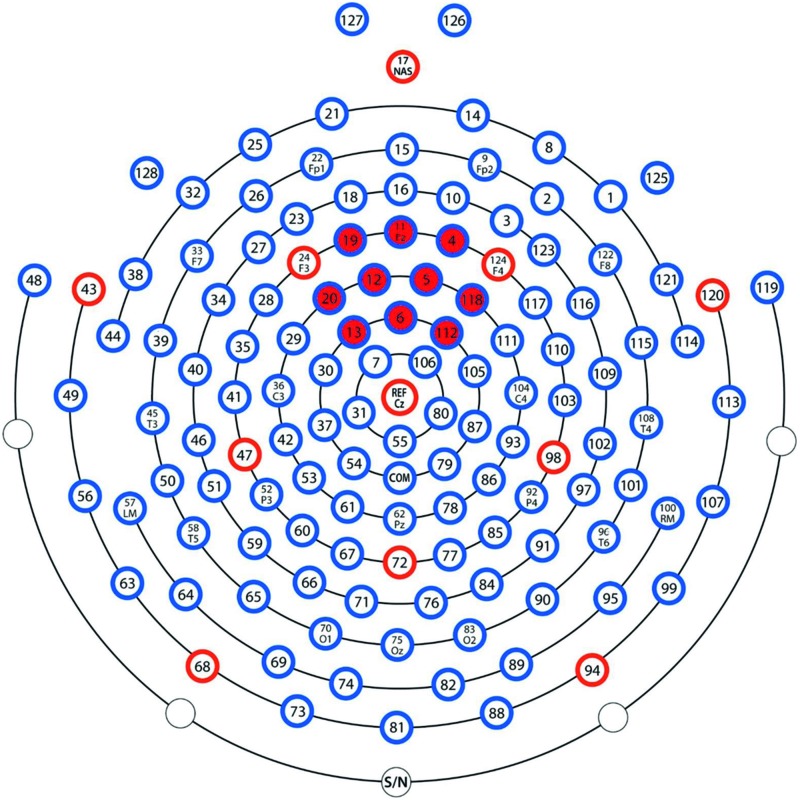
**Map covering leads used in analysis (the ones used in the analysis are marked red)**.

The Nc is commonly found at central or fronto-central leads at around 300–600 ms post stimulus (Wiebe et al., 2006; Burden et al., 2007; de Haan, 2007; Riggins et al., 2013). In the present study, Nc was defined as the peak in mean amplitude from a cluster of 10 leads situated around the Fz lead (leads 4, 5, 6, 11, 12, 13, 19, 20, 112, and 118) between 300 and 600 ms post stimulus. See map over leads used in **Figure 3**. To build upon the previous findings (Heimann et al., 2013) and to get an index of learning of the presented picture-pairs, comparisons between the first and the last presentation of the LP as well as between the last presentation in the LP and both the test phases were performed by calculating the change in Nc amplitude between these presentations. We expected the Nc to decrease with repetition during the LP, and increase when unexpected pairs were presented in the test phases.

#### Statistical Analyses

SPSS version 21 was used to analyze data in the current study. For comparison between Nc mean amplitudes at the beginning and the end of the LP, the second presentation of the LP was compared to the final repetition in the LP using Student’s *t*-test. For comparison between Nc mean amplitudes at LP and test phase, repeated measures ANOVA was used, with three factors including phase; Pres 5, Recomb, and Novel. Pearson correlation was conducted in order to test relationships between measures of memory, communication and associative memory. One-tailed results were used for our specific predictions; all other comparisons and correlations are two-tailed.

## Results

### Behavioral Measures

#### Deferred Imitation

The results for DI are displayed in **Table 1**. For the brief memory delay (30 min) the participants performed significantly more target actions than the comparison group who had not seen the actions previously. In contrast, no indication of any retained memory was observed for the long memory delay. Furthermore, there was no significant correlation between memory performance after the brief and the long delays, *r_s_*(8) = 0.365, *p* = 0.374 for the experimental group.

**Table 1:** Mean and range for deferred imitation (DI) with a brief delay (DI brief) and with a long delay (DI long) for the Experimental and Comparison groups.

	Experimental group				Comparison group			
	*M*	SD	Range	*N*	*M*	SD	Range	*N*
DI brief	2.13*	0.96	0–3	16	0.88	0.84	0–2	8
DI long	0.38	0.52	0–1	8	0.38	0.52	0–1	8

*p < 0.05 compared to comparison group.

#### Communication

Parental ratings of the participants’ communicative development are shown in **Table 2**. Of the 14 participants for whom ERP data exists, 11 performed above the 10th percentile (31.5) for their age on receptive language, according to Eriksson and Berglund (1999). The 10th percentile cutoff was chosen in order to include only children that had come somewhat along in their language development.

**Table 2:** Mean frequency of words and range for communicative development based on the Swedish Early Communicative Development Inventories (SECDI) ratings for the Experimental group.

Measure/part of SECDI	*M*	SD	Range	*N*
Receptive language	82.00	60.88	10–201	14
Productive language	9.86	9.92	0–35	14
Gestural development	34.43	11.24	12–54	14

### Electrophysiological Measures

#### Associative Memory

The mean amplitudes for each phase/condition are specified in **Table 3**.

**Table 3:** Mean negative central (Nc) amplitudes of learning and test phase conditions.

Phase	Mean Nc amplitude	SD
**Learning phase (LP)**
Pres 1^a^	-10.07	5.71
Pres 2^a^	-12.80	8.46
Pres 3^a^	-9.43	7.82
Pres 4^a^	-10.80	6.31
Pres 5^a^	-8.52	8.09
Reminder^b^	-10.83	7.47
**Test phase**
Recomb^c^	-11.73	6.58
Novel^d^	-12.51	6.79

*^a^Pres, Presentation 1–5 of the picture-pairs of the LP.*

*^b^Reminder of LP. Excluding one outlier who obtained >3 SD, *n* = 15.*

*^c^Recomb, recombination of picture-pairs.*

*^d^Novel, combination of one familiar and one novel picture.*

**FIGURE 4:**
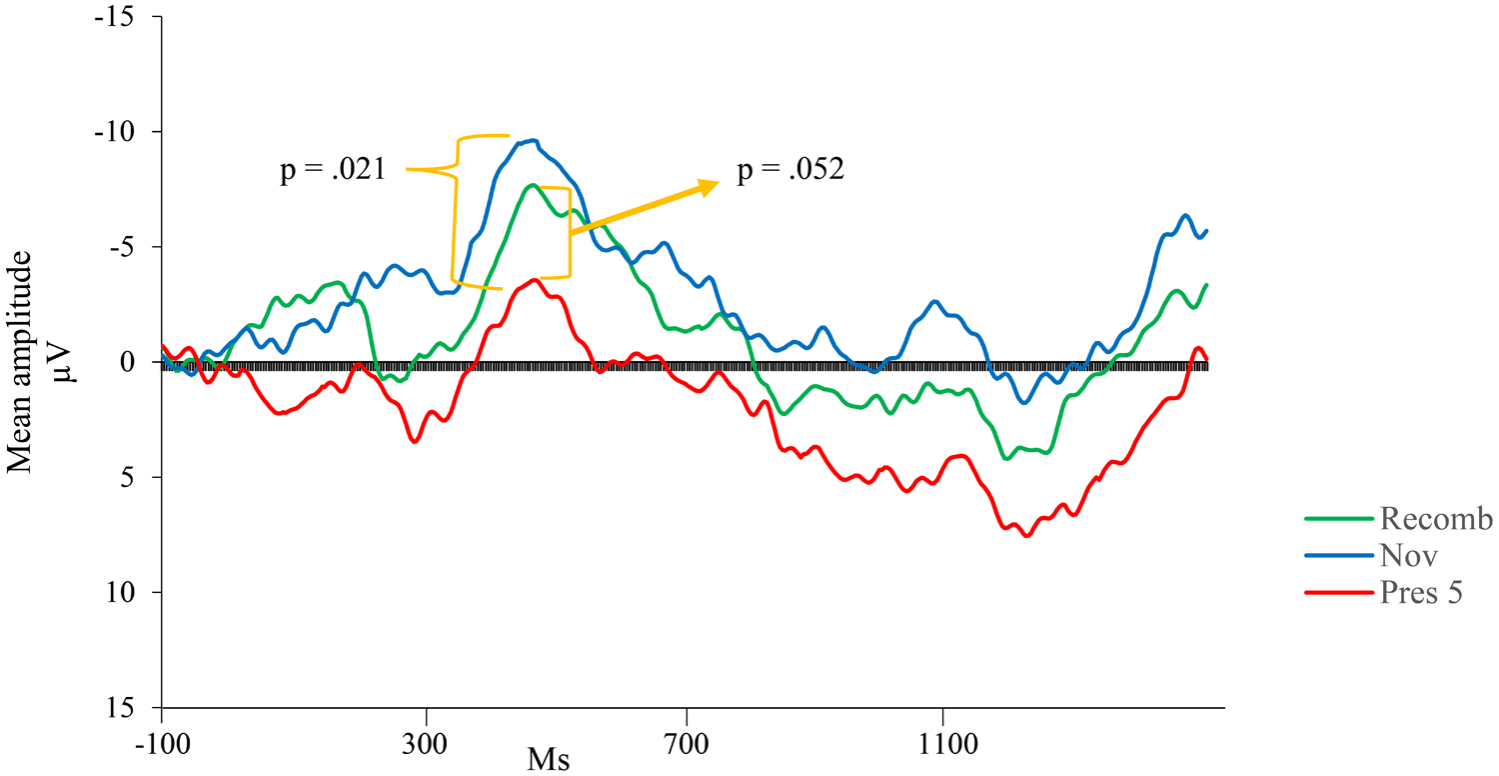
**Group mean event-related potentials (ERPs) for the last presentation in the LP (Pres 5) and the two variations of the test phase; Nov (combination of one familiar picture and a novel one), and Recomb (recombination of pictures)**.

Visual inspection revealed a peak of negative activity within the Nc time window (400–600 ms post stimulus; see ). A comparison between the test phases (Recomb and Novel) and the last presentation of the LP (Pres 5) was conducted for the Nc component. A repeated measures ANOVA with three factors including condition (Pres 5, Recomb, and Novel) showed a significant effect of phase, *F*(2,30) = 3.454, *p* = 0.045, $\eta_{p}^{2}$ = 0.19. Pairwise comparisons using Fisher’s LSD *post hoc* test revealed that Pres 5 differed significantly from Nov; Mean difference = 4.20, *p* = 0.021, one-tailed, in accordance with our prediction and there was also a tendency toward the predicted significant difference between Pres 5 and Recomb; Mean difference = 3.21, *p* = 0.052, one-tailed. That is, for Novel, the mean amplitude of the Nc was significantly more negative than at the end of the LP. However, there was no significant difference between the two test phases, Recomb and Novel; Mean difference = 0.993, *p* = 0.41. See overview in ** Figure 4**.

In order to further investigate learning of repetitions, a comparison was made between the initial LP (Pres 1, where the picture pairs are first presented; and Pres 2, where the actual repetition of picture-pairs begins) and the final presentation in the LP (Pres 5). Results reveal that for the comparison between Pres 1 and Pres 5, there was no significant difference (*p* = 0.243, one-tailed). However, there was a tendency toward a significant decrease in amplitude between Pres 2 and Pres 5, *t*(15) = -1.549, *p* = 0.07 (one-tailed), *d* = 0.52, indicating a tendency to learning as expected.

**Table 4** shows that the expected pattern of ERP’s was obtained for 10 out of 16 participants, i.e., that the negativity of the mean amplitude of the Nc component decreased during the LP and increased again between the learning and test phases.

**Table 4:** Change in mean amplitude within the LP and between the end of the LP and the test phase conditions (Novel and Recomb).

	Learning phase		Test phase			
			Novel		Recomb	
	*N*	Proportion	*N*	Proportion	*N*	Proportion
Expected	10	0.62	11	0.69	9	0.56
Not expected	6	0.37	4	0.25	3	0.19
No change*	0	0	1	0.06	4	0.25

*No change between the last presentation on the LP and test phases = 0 ± 1μV.

For the Novel condition, the negativity of the Nc mean amplitude for 11 out of 16 participants (69%) increased as expected in comparison to the last presentation of the LP. For four participants (25%) there was a decrease and for one participant there was no change in mean amplitude.

The comparison between Recomb and the last presentation of the LP revealed that 9 of 16 participants (56%) showed the expected increase in negativity of the Nc mean amplitude, three showed response decrease and four showed no change at all.

In addition, 12 out of 16 participants demonstrated the same (expected) pattern of increase in negativity between the last presentation of the LP and both Novel and Recomb.

### Relationships Between Behavioral and Electrophysiological Measures

#### Deferred Imitation

Since DI with a long delay was unsuccessful (no memories were retained), only results pertaining to DI with a brief delay are presented.

There was a significant positive correlation with the change in mean amplitude of Nc between Pres 5 and Novel, *r*(15) = 0.566; *p* = 0.011, one-tailed; but the correlation did not reach significance with the change between Pres 5 and Recomb, *r*(15) = 0.328; *p* = 0.108, one-tailed. A positive correlation means here that a high score on DI is associated with change in the non-expected direction (i.e., positive or small/no negative change in mean amplitude between learning and test phases).

A visual inspection of the scatter plot indicates the possibility of two different response patterns. The seven participants who achieved a maximum score of three on DI had an Nc that did not change as expected; there was almost no amplitude change between the learning and test phases (*M* = -0.86). In contrast, the nine children with an imperfect memory score (*M* = 1.18) did show the expected pattern; the mean amplitude in Nc changed substantially from the LP (*M* = -6.44) to the test phase (*M* = -15.35).

**FIGURE 5:**
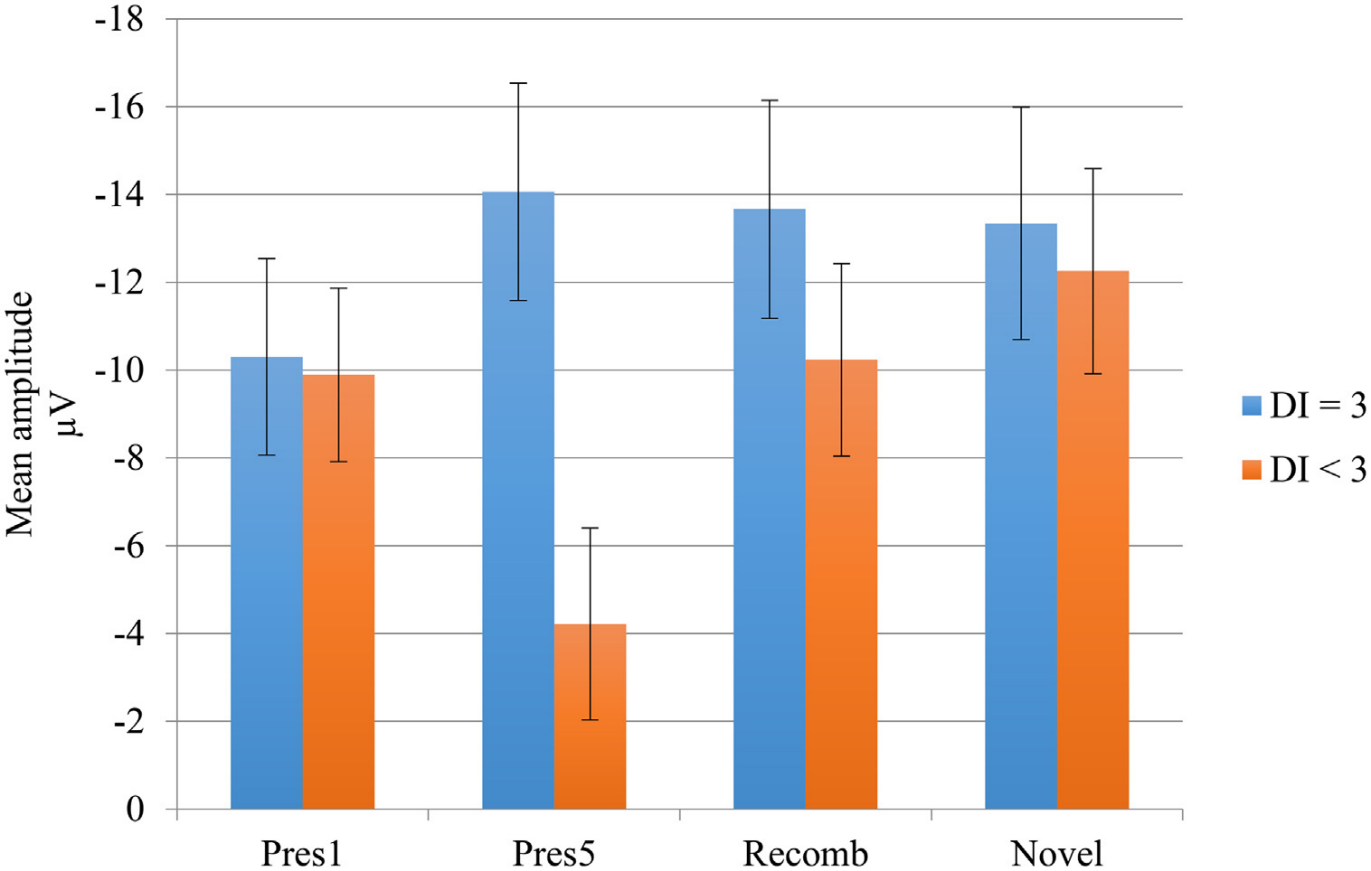
**Negative central (Nc) mean amplitudes of the LP and test phases for the deferred imitation (DI) subgroups.** Subgroup “DI = 3” includes the seven children with maximum score on DI, and subgroup “DI < 3” includes the rest of the children (*n* = 9), i.e., those performing 0, 1, or 2 actions.

To test this difference statistically, we divided the group according to their performance on DI with a brief delay. The participants who obtained maximum score constituted one subgroup (*n* = 7) while all the others constituted a second group (*n* = 9). We entered the subgroups into two ANOVAs investigating the difference in mean amplitude between the end of the LP and the two test phases. The analysis including Novel revealed a significant main effect of phase, *F*(1,14) = 5.24, *p* = 0.038, $\eta_{p}^{2}$ = 0.27 showing greater negativity for the Novel test phase than at the end of the LP. The main effect of group did not quite reach significance, *F*(1,14) = 3,26, *p =*0.092, $\eta_{p}^{2}$ = 0.19. However, there was a significant interaction, *F*(1,14) = 7.52, *p* = 0.016, $\eta_{p}^{2}$ = 0.35, showing that the change in mean amplitude between LP and test phase was attributable to the subgroup with the lower performance on DI. The simple main effects of phase were calculated for this group; for Novel; *t*(8) = 3.677, *p* = 0.006; and for Recomb; *t*(8) = 2.327, *p =*0.048, revealing a significant difference between Pres 5 and both of the test phases. There was no significant main effect of phase, *F*(1,14) = 2.63, *p =*0.13, $\eta_{p}^{2}$ = 0.16, in the ANOVA including Recomb but there was a significant main effect of group, *F*(1,14) = 5.5, *p* = 0.034, $\eta_{p}^{2}$ = 0.28, and the interaction almost reached significance, *F*(1,14) = 3.42, *p =*0.085, $\eta_{p}^{2}$ = 0.19. The results are illustrated in **Figure 5**.

#### Communication

Using all available data (*n* = 14) we found no significant correlations between DI with a brief delay and any of the communicative measures. However, including only those participants who performed above the 10th percentile (*n* = 11), the comparison between the SECDI subscale “understanding words” measuring receptive language and the change in Nc mean amplitude between the end of the LP (Pres 5) and the test phase revealed a significant negative correlation both with Recomb, *r*(11) = -0.603, *p* = 0.050, and Novel, *r*(11) = -0.638, *p* = 0.035. This means that the participants who showed an increased Nc amplitude had a higher score on the receptive language part of SECDI. The other two aspects of early communicative development measured by SECDI, productive language and gestural development, did not correlate significantly with the change in mean Nc amplitude for Recomb or for Novel. See **Table 5** for all correlations.

**Table 5:** Correlations between event-related potential change scores, DI (brief delay), and receptive language (SECDI).

ERP change score			Correlations			
	*M* (*n* = 16)	SD	DI (*n* = 16)		Receptive language (*n* = 11)^3^	
			*r*	*p=*	*r*	*p=*
Nc Recomb – Pres 5	-3.21	7.41	0.328	0.107^1^	-0.603	0.050^2^
Nc Novel – Pres 5	-4.20	7.60	0.566	0.011^1^	-0.638	0.035^2^

*^1^One-tailed.*

*^2^Two-tailed.*

*^3^For those performing above the 10th percentile.*

## Discussion

The results of the present study provide partial support for our hypotheses: for 14-months-old children there is an association between early declarative memory as reflected by deferred imitation and associative memory as reflected by electrophysiological measures. We propose that the basis for this association is the ability to form, maintain, and recall mnemonic respresentations.

Our first prediction was that there would be an increase in the negativity of the mean amplitude of Nc between the end of the LP and both test phases, indicating a response not only to novelty but also to violation of expectations of learned associations, indicating associative memory. This expectation was only partly supported by our results since the increase was significant only for those participants who did not achieve ceiling performance on DI, and only for the Novel condition. Here, there was a significant difference between the end of the LP and Novel. However, this was not the case for the participants obtaining maximum scores on DI. We have at present no straightforward explanation of this response pattern but we speculate that the two patterns may either reflect differences in how fast the children’s memory is consolidated or that the responses elicited by our ERP paradigm reflected a process other than the one we expected, based on the setup of our paradigm. This is something future studies need to explore further, our observations can be taken as indicative at most.

The general pattern of findings is in line with our earlier study (Heimann et al., 2013) in that we can report significant links between behavioral measures of memory and associative memory measured through ERP. However, on a more detailed level, it differs partly from our previous results. Heimann et al. (2013) reported significant change in Nc mean amplitude for the picture-pairs in new combination in comparison to the LP (assumed to be a stronger indicator of associative memory; e.g., Aggleton et al., 2012) and not for the novel combination while we now find significant results primarily for the novel combination. However, since the predicted finding for the recombined combination came close to significance (*p* = 0.052) we are reluctant to completely rule out the possibility that the findings reported here also reflect associative memory according to a more stringent definition.

We found no indication of memory of the actions included in the DI after a long delay although previous work has shown that 14 months-old children can form representations that enable them to remember after a delay of up to 4 months (Meltzoff, 1995). One reason for this might be that our tasks are probably more difficult than the ones used by Meltzoff, since we included two “odd” tasks such as using the elbow to fold a hinge and a pen to push a button. In addition, the number of participants differed: eight in our study as compared with 48 in Meltzoff’s study.

In the present study we also explored the relationship between ERP and early communication. Significant correlations were found between associative memory and receptive but not expressive language measures. To our knowledge, this is the first time a relationship between a neural index of associative memory and parental ratings of receptive language has been found in this age group. The correlation between receptive language and change in mean Nc amplitude was negative. That is, the participants who had better associative memory, also understood more words. This might suggest a common basis for picture association and word-to-meaning association, possibly mediated by the episodic buffer of working memory (Rudner and Rönnberg, 2008). However, due to the small *n* (11) in the SECDI-ERP correlation, the interpretation of this result is to be made with caution. In comparison, Torkildsen et al. (2008) reported a relationship between parental ratings of productive language development and neural correlates of associative memory as reflected by word-to-picture association (N400) in 20 months-old children. We did not find this kind of relationship for parental ratings of productive language, which could be due to age-related differences in language skills between the samples included in our and Torkildsen et al.’s (2008) study. The children in Torkildsen et al.’s (2008) are likely to have been much further along in their language development compared to our 14 months-old children. However, in the present study, associative memory as reflected by ERP correlated with vocabulary, suggesting that children with a larger vocabulary might have a more efficient encoding. This might be interpreted as being partly in line with suggestions put forward by Gathercole et al. (1999) and Ullman (2004), i.e., that there is a connection between memory and language development.

Limitations of this study concern foremost the large attrition clearly affecting the power of the results. Initially 44 participants were included in the study and of these 35 children accepted to wear the electrode cap. Of these 35, only 16 children provided acceptable ERP data due to various reasons such as recordings containing excessive amounts of artifacts or children not completing enough trials. An attrition rate around 50% is not unusual for infant ERP data (e.g., Bauer et al., 2006; Snyder et al., 2010; Stets et al., 2012). A second limitation that also concerns the ERP data is that it could be argued that including only eight trials in the average is risking less reliability of the ERP data, many studies sets the limit to 10 or more (see for example Luck, 2005; de Haan, 2007; Leppänen et al., 2007). However, it has been argued by Stets and Reid (2011) that a limit of 10 trials is usually based on adult data and that this amount of trials is seldom obtainable from infants. Thus, we decided to include fewer trials than in our previous study and we manually re-analyzed the whole data set to make sure that no trials that contained any artifacts were included. In essence, we aimed for fewer but cleaner trials in order to increase the quality of our ERP data.

The small subsample (*n* = 8) participating in the DI condition with a longer delay is an additional limitation of the study. The same goes for the measures of language and communication; for two of the participants the questionnaires were not returned or were not completely filled in, resulting in loss of data. Although half of the participants took part in our previous study it should be underscored that the ERP data was re-analyzed and is thus not comparable with previously reported findings. The only reused data not reanalysed are the results for DI for these eight children.

More research combining behavioral measures and electrophysiological measures of cognition in prelingual infants is needed, since a combination of measures serves as an important tool in the search of a more fine-grained description of the processes underlying memory development. Future studies should use larger samples and refined analysis methods in order to validate the present results. In addition, longitudinal studies would generate essential information on the stability of the reported relationships between various types of early memory and language development.

In spite of the limitations, we believe that by combining electrophysiological and behavioral measures, as done in the present study, important steps toward a better understanding of early learning and memory development have been taken. Our results demonstrate that a relation exists in 14-months-old children between electrophysiological measures of associative memory and behavioral measures of early declarative memory, as well as between associative memory and vocabulary.

## Author contributions

This study was designed by MH, MJ, and ML. Data were acquired by EN and MH and analyzed by EN. All authors were involved in interpretation of results. The first draft of the manuscript was prepared by EN. All authors took part in critical revision of the manuscript.

## Conflict of Interest Statement

The authors declare that the research was conducted in the absence of any commercial or financial relationships that could be construed as a potential conflict of interest.
